# Supplementary material for: Structural Basis of Mutation-Dependent p53 Tetramerization Deficiency
Source: Int J Mol Sci. 2022 Jul 19;23(14):7960. doi: 10.3390/ijms23147960 (PMC9316806; doi:10.3390/ijms23147960)
Supplement: Supplementary file 1 [file ijms-23-07960-s001.zip › ijms-1815171-supplementary.pdf]

International Journal of
Molecular Sciences

MDPI

Article

Structural Basis of Mutation-Dependent p53 Tetramerization Deficiency

Marta Rigoli, Giovanni Spagnolli, Giulia Lorengo, Paola Monti, Raffaello Potestio, Emiliano Biasini and Alberto Inga

Supplementary Figures and Figure Legends

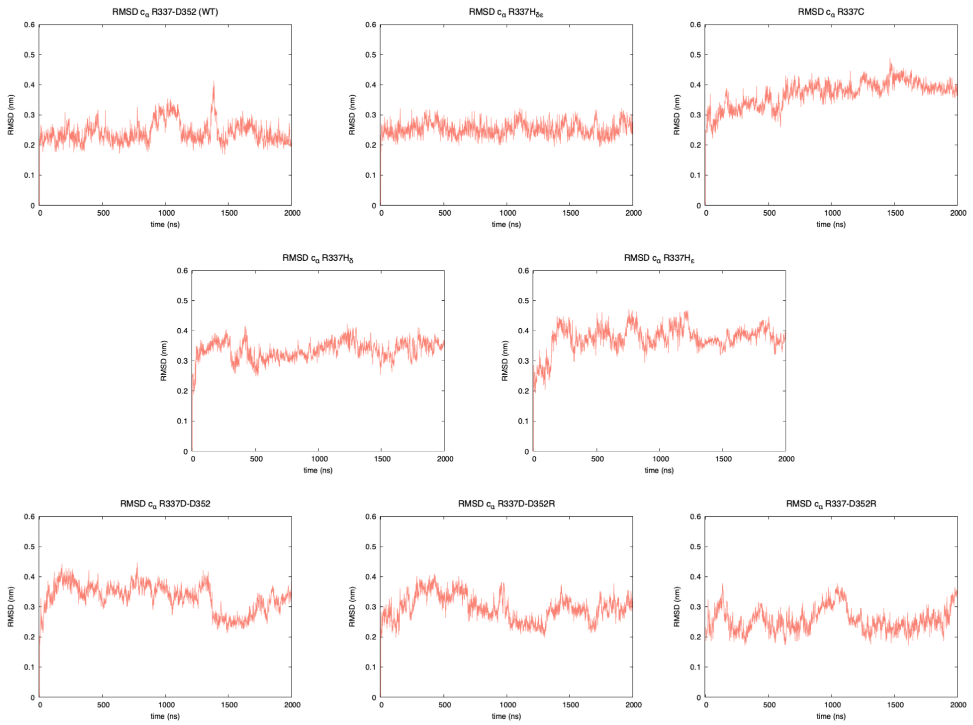

Figure S1. Convergence of the MD trajectories. Values of RMSD as a function of time are plotted for the WT protein and all the mutated forms. Each form rapidly reaches the convergence to structural equilibrium in less than 200 ns.

Citation: Rigoli, M.; Spagnolli, G.; Lorengo, G.; Monti, P.; Potestio, R.; Biasini, E.; Inga, A. Structural Basis of Mutation-Dependent p53 Tetramerization Deficiency. *Int. J. Mol. Sci.* **2022**, *23*, 7960. https://doi.org/10.3390/ijms23147960

Academic Editor: Masayuki Takahashi

Received: 28 June 2022
Accepted: 15 July 2022
Published: 19 July 2022

Publisher's Note: MDPI stays neutral with regard to jurisdictional claims in published maps and institutional affiliations affiliations.

Copyright: © 2022 by the authors. Submitted for possible open access publication under the terms and conditions of the Creative Commons Attribution (CC BY) license (https://creativecommons.org/licenses/by/4.0/).

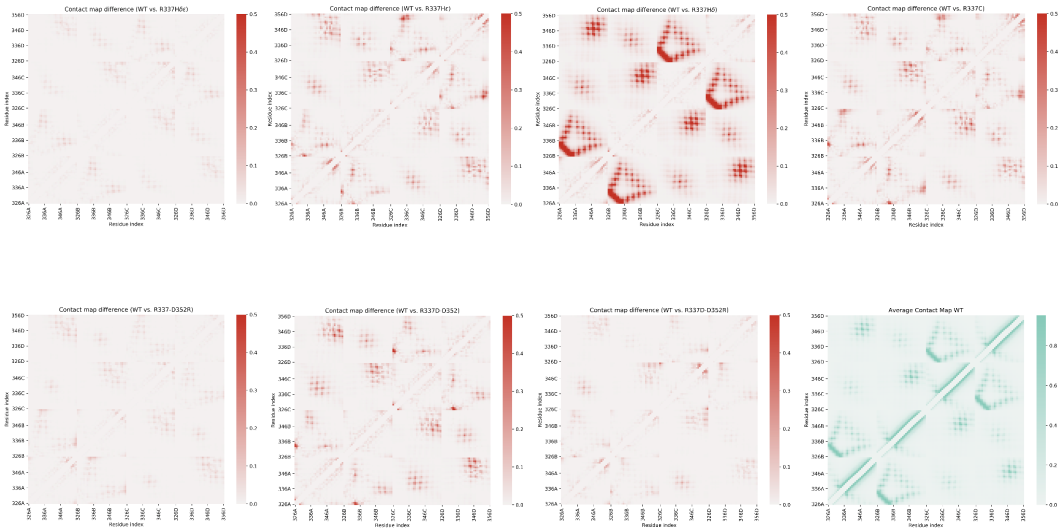

Figure S2. Contact map analysis of WT and mutant p53 TET domain. Each box shows the average contact map from the different trajectories of the indicated p53 molecules, plotted as a residue index matrix. Mutant p53 forms are expressed as the absolute value of the difference with the WT protein (lower-right corner). The contact maps of R337C, R337D/D352R, R337D, R337H$_{\varepsilon}$, and R337H$_{\delta}$ show significant variations, with the latter presenting the largest divergence. In contrast, D352R and R337H$_{\delta\varepsilon}$ display no substantial deviation from the WT. A threshold of 0.5 was chosen as the cut-off.

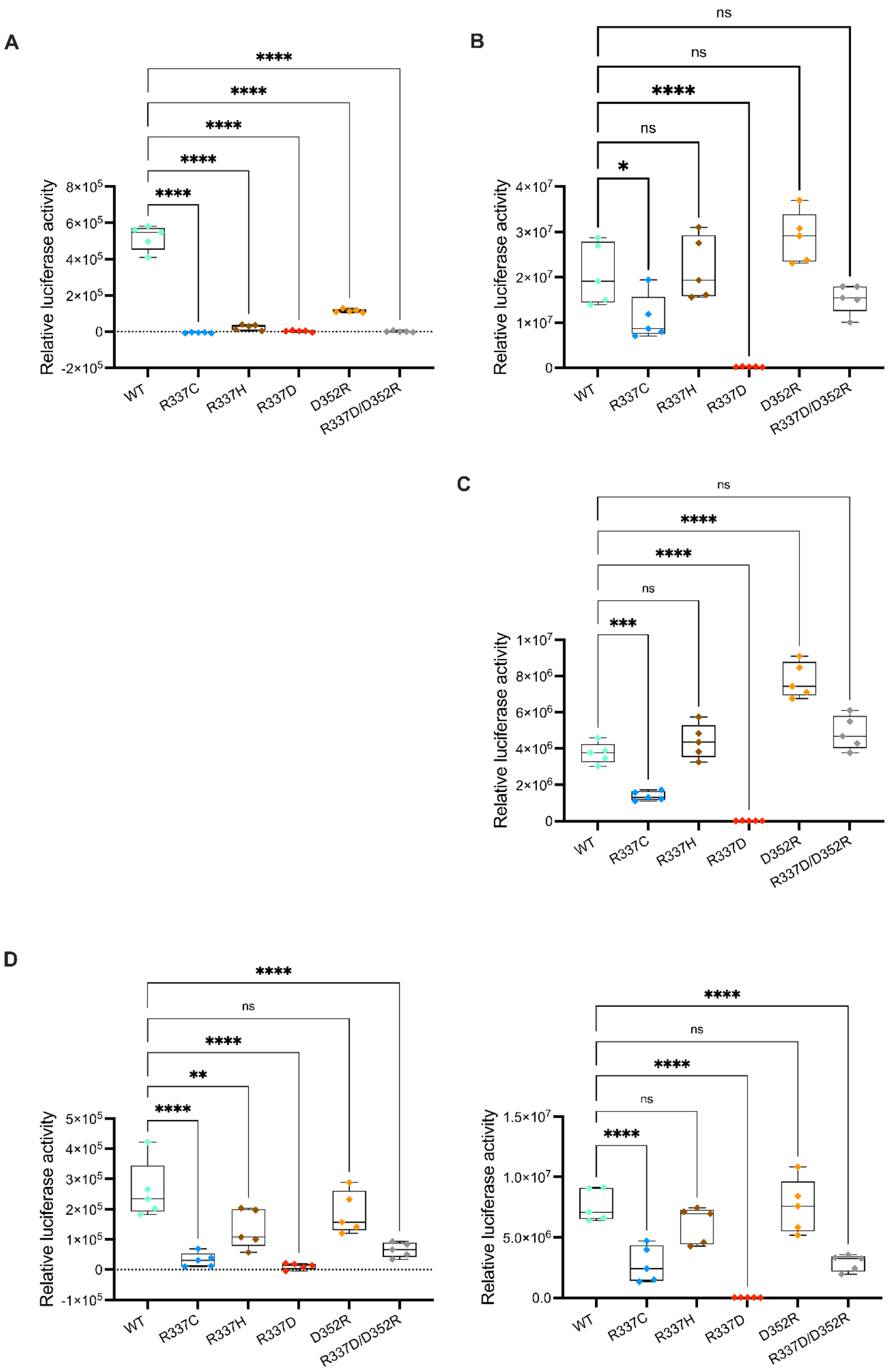

Figure S3. Transactivation potential of p53 TET mutations at different levels of p53 expression. Luciferase assays were performed as described in Figure 5 using the indicated reporter strains that are isogenic except for the sequence of the p53 response element that allows p53 to bind to the promoter region upstream of the *Photinus* pyralis reporter and stimulate its transcription. A. The p21-5' RE strain is highly responsive to p53. It can

register p53-induced transactivation even in cells resuspended directly from glucose plates where the *GAL1* promoter, which controls p53 expression, is at its lowest level of transcription. Using this condition, the difference between p53 alleles is more visible, as the p53 protein levels are limiting. See Figure 5 for a comparison. B. Culturing cells in media containing higher levels of galactose (0.032%) stimulates the expression of the luciferase reporter in a p53-dependent manner. However, the difference in activity among the alleles is reduced or no longer appreciable. This is particularly evident with the R337H and D352R in the p21-5' reporter strain and R337H and the double mutant R337D/D352R in the p21-SP2 strain, while D352R continued to exhibit higher activity compared to WT p53. C. The reporter strain derived with the spaced p21-5' RE was tested at high galactose (0.128%). The higher relative activity of D352R was confirmed. D. To investigate if the effect of D352R was dependent on a generally lower affinity of p53 for its binding sites or more directly to the structural arrangement of RE subunits, we also tested a lower affinity p53 RE derived from the PUMA promoter. In this case, the lower transactivation potential is related to mismatches from the consensus sequence and not to the presence of a spacer between the decameric half-sites. Left and right panels plot data obtained from cultures exposed to 0.008% or 0.032% galactose for six hours, respectively. Results were processed and are plotted as described in Figure 5. * $p<0.05$; ** $p<0.01$; *** $p<0.001$; **** $p<0.0001$, ns = not significant; one-way ANOVA with Dunnett's multiple comparison test.

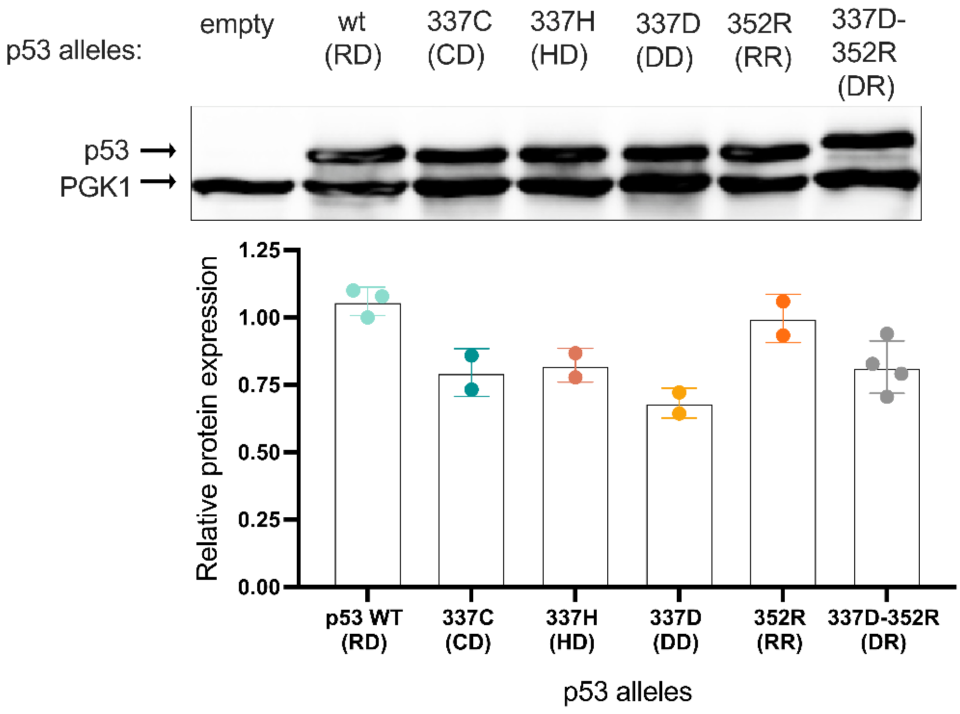

Figure S4. p53 TET mutants are expressed at similar levels in the yeast reporter strains. Representative western blot (top panel) and quantification of the densitometric scan of the immunodetection comparing the relative expression of WT p53 and the various mutant alleles tested in this study. PGK1 was used as a reference protein. All the p53 tetramerization mutants were expressed at similar levels. We noted a slight decrease in migration in the SDS-PAGE for the double mutant R337D/D352R.
